# Effects of a less invasive suction protocol on pain and physiological stability in preterm infants receiving non-ınvasive ventilation: a randomized controlled trial

**DOI:** 10.1186/s12887-026-07212-8

**Published:** 2026-06-26

**Authors:** Ebru Temizsoy, Sevilay Topcuoglu, Havva Aliustaoglu, Esra Ozkan, Guner Karatekin

**Affiliations:** 1 Istanbul Bilgi University, Istanbul, Turkey; 2 University of Health Sciences, Zeynep Kamil Women and Children Diseases Training and Research Hospital, Istanbul, Turkey; 3 Mersin City Hospital, Mersin, Turkey

**Keywords:** Non-invasive ventilation, Nasopharyngeal suction, Premature infants, Procedural pain, Physiological monitoring, Randomized controlled trial

## Abstract

**Background:**

Non-invasive ventilation (NIV) is widely used in preterm infants to reduce ventilator-associated lung injury. However, nasopharyngeal suctioning during NIV is an invasive procedure that may cause pain, stress and physiological instability in preterm infants. This study aimed to compare the effects of less invasive suction protocol and conventional nasopharyngeal suctioning on pain and physiological stability in preterm infants receiving non-invasive ventilation.

**Methods:**

This randomized controlled trial was conducted in a tertiary neonatal intensive care unit. Preterm infants with a gestational age of 32^0/7^ to 36^6/7^ weeks who required NIV were eligible. A total of 100 infants were randomly assigned to either the less invasive suction protocol group (*n* = 50) or the conventional suction group (*n* = 50). Pain was assessed using the Neonatal Pain, Agitation and Sedation Scale (N-PASS). Heart rate (HR), respiratory rate (RR), oxygen saturation (SpO₂), and skin color were recorded before suctioning, during the procedure, and 10 min after completion. Between-group comparisons were performed using appropriate parametric or non-parametric statistical tests, with *p* < 0.05 considered statistically significant.

**Results:**

Baseline demographic and clinical characteristics were comparable between groups. During suctioning, infants in the less invasive suction protocol group had significantly lower HR and higher SpO₂ levels compared to the conventional group (*p* < 0.05). The proportion of infants with pink skin color was significantly higher in the less invasive suction protocol group. N-PASS pain scores during the procedure were significantly lower in the less invasive suction protocol group (*p* < 0.05). No significant differences were observed between groups in RR measurements at any time point.

**Conclusions:**

The less invasive suction protocol reduced procedural pain and supported physiological stability in preterm infants receiving NIV. Minimizing airway manipulation may contribute to safer and more developmentally supportive care in neonatal intensive care units.

**Trial registration:**

ClinicalTrials.gov (NCT07111611), registered on 08 August 2025 (retrospectively registered).

**Supplementary Information:**

The online version contains supplementary material available at https://doi.org/10.1186/s12887-026-07212-8.

## Background

Non-invasive ventilation (NIV) is one of the primary respiratory support strategies aimed at reducing ventilator-associated lung injury in preterm infants [1]. However, airway secretion management in neonates receiving NIV remains a significant clinical challenge. Accumulated secretions may compromise airway patency, increase the work of breathing, and impair oxygenation, thereby reducing the effectiveness of NIV [1, 2].

Although airway management in preterm infants is a multidisciplinary responsibility, secretion assessment and suctioning during NIV are largely performed by nurses. Key parameters such as catheter selection, negative pressure, duration, and frequency of suctioning vary in clinical practice, often due to limited evidence and reliance on clinical experience [3]. While randomized studies have emphasized the importance of optimizing suction practices to improve outcomes in mechanically ventilated patients [4], evidence specific to neonatal populations remains limited. Current guidelines recommend need-based suctioning to minimize trauma and maintain physiological stability [5]; however, variability in clinical decision-making continues to result in inconsistent practices [6].

Nasopharyngeal suctioning is a frequently performed intervention in preterm infants receiving NIV; however, it is not without risk. Airway manipulation during suctioning has been associated with bradycardia, oxygen desaturation, and cardiorespiratory instability [5, 7]. In addition, suctioning is a significant source of procedural pain in preterm infants. Due to immature autonomic regulatory mechanisms, these infants may exhibit exaggerated and prolonged physiological responses, including changes in heart rate and oxygen saturation [8, 9].

Studies indicate that a substantial proportion of procedures performed in NICUs are associated with moderate to severe pain and that preterm infants are exposed to numerous painful interventions during hospitalization [10, 11]. Repeated exposure to procedural pain has been associated not only with acute physiological instability but also with alterations in brain development and long-term neurodevelopmental outcomes [8, 12–14]. Therefore, reducing pain associated with frequently performed interventions remains a clinical priority. Various non-pharmacological interventions have been investigated to reduce suction-related pain in preterm infants. Methods such as swaddling, oropharyngeal colostrum administration, and auricular massage have demonstrated beneficial effects on procedural pain [15]. Nevertheless, these approaches mainly provide supportive care and do not directly target the traumatic component of the suction technique. Recent evidence highlights the importance of incorporating strategies that minimize procedural trauma as part of neonatal pain management [16].

Preterm infants in neonatal intensive care units are frequently exposed to painful procedures that may lead to physiological instability [8, 10, 14]. Suctioning remains an essential component of airway management in infants receiving NIV due to the risk of secretion accumulation and airway obstruction [1, 2]. However, unnecessary suctioning may cause harm, highlighting the importance of optimizing suction techniques rather than eliminating the procedure [5].

Recommendations aimed at reducing mucosal trauma are limited in the literature. Nasopharyngeal irrigation with saline has been suggested to reduce the need for catheter insertion [17], and less invasive suction techniques allowing aspiration without direct mucosal contact have also been proposed [18]. These approaches aim to minimize catheter advancement and mechanical airway stimulation, thereby reducing physiological instability and pain response. However, these techniques have not been systematically evaluated in randomized controlled trials. To date, no randomized controlled trial has directly addressed the traumatic component of suction techniques in preterm infants receiving NIV.

Given the vulnerability of preterm infants to cardiorespiratory fluctuations and autonomic stress responses, the development and evaluation of less traumatic suction techniques remain clinically important for both short-term stability and long-term neurodevelopmental outcomes. The aim of this study was to compare the effects of less invasive suction protocol and conventional nasopharyngeal suctioning on pain and physiological stability in preterm infants receiving non-invasive ventilation.

## Methods

### Study design and setting

This prospective, randomized controlled clinical trial was conducted to evaluate the physiological, clinical, and behavioral effects of a less invasive suction technique in preterm infants receiving non-invasive ventilation (NIV). The study was carried out in the Level IV Neonatal Intensive Care Unit (NICU) of Zeynep Kamil Women’s and Children’s Diseases Training and Research Hospital, Istanbul, Turkey, between January 2018 and January 2020.

### Participants, sample size, and randomization

Preterm infants born at 32^0^/^7^–36^6^/^7^ weeks of gestation who were receiving nasal continuous positive airway pressure (nCPAP) support during hospitalization in the NICU between January 2018 and January 2020 were eligible for inclusion. No other modes of non-invasive ventilation were used. Infants meeting the inclusion criteria and whose parents provided written informed consent were enrolled.

Sample size calculation was performed using G*Power 3.1.9.2 software based on the mean difference and standard deviation of N-PASS pain scores obtained from a pilot study. Assuming a two-independent group comparison, α = 0.05 and power (1 − β) = 0.90, a minimum of 45 neonates per group was required. Considering potential dropouts, 50 neonates were included in each group (total* n* = 100).

Randomization was performed using the urn method, which is considered equivalent to complete randomization. In this method, two types of balls (black and white) were used to represent group allocation. One ball was randomly selected for each allocation; if black, the infant was assigned to one group, and if white, to the other group. In this study, the white ball represented the intervention group and the black ball represented the control group. The balls were placed in an opaque bag prepared by the researcher. For each eligible infant, a person not involved in the study selected a ball blindly to ensure allocation concealment. After each allocation, the ball was returned to the bag before the next selection.

### Data collection procedure

Written and verbal informed consent was obtained from the parents prior to the suction procedure. Data were collected by trained research nurses working in both day and night shifts. Standardized data collection forms were completed for each neonate to record demographic and clinical characteristics. Suctioning was not performed at predetermined intervals. The need for suctioning was determined based on the presence of secretions and clinical assessment.

Physiological and behavioral parameters were recorded at three time points: before suction, during suction, and 10 min after suction. Suctioning was performed only in preterm infants considered physiologically stable. All necessary materials were prepared prior to the procedure, and infants were placed in the supine position.

Heart rate, respiratory rate, oxygen saturation (SpO_2_), skin color, and N-PASS pain and sedation scores were recorded before suction.

Pain and physiological assessments were performed by three trained research nurses using standardized assessment protocols to ensure consistency in measurements. Due to the nature of the intervention and the clinical setting, blinding was not feasible. Data analysis was conducted using coded group assignments.

### Intervention procedure (less invasive suction protocol)

In the intervention group, suctioning was performed using a less invasive suction protocol. A modified 10 Fr suction catheter with the distal tip cut and a 10 mL syringe were used. During each suction procedure, sterile 0.9% sodium chloride was administered into one nostril in small aliquots to facilitate secretion mobilization, consistent with previously described techniques in the literature (Waisman, 2006). When necessary, repeated small-volume administrations were performed based on the infant’s clinical condition, with a total volume typically not exceeding 5–10 mL. The solution was not administered as a single bolus.

Simultaneously with saline instillation, secretions were aspirated from the opposite nostril and oral cavity using a negative pressure of approximately 150 mmHg. Care was taken to avoid direct mucosal contact to minimize mucosal trauma and no pressure was applied during saline instillation. The suction procedure was completed within 5–10 s. Physiological and behavioral parameters were recorded during the procedure. Care was taken to ensure that the syringe tip was loosely positioned within the nostril.

### Control procedure (conventional suction)

In the control group, suctioning was performed according to routine clinical practice. Saline was not routinely used. Suctioning was performed using 6–8 Fr suction catheters via the oral cavity and, when necessary, the nostrils, with a negative pressure of 60–100 mmHg for 5–10 s. Physiological and behavioral parameters were recorded during and after the procedure.

### Data collection forms and tools

Two standardized forms were used. The descriptive characteristics form included sex, gestational age, postnatal age, birth weight, duration of NIV, day and duration of nasal continuous positive airway pressure (nCPAP), number of suction procedures (daily and total), and length of hospital stay.

The follow-up form included date and time of suction and physiological parameters (HR, RR, SpO_2_, skin color) and behavioral parameters (N-PASS pain and sedation scores).

All measurements were recorded at baseline, during suction, and 10 min after suction. Pain and sedation were assessed using the Neonatal Pain, Agitation and Sedation Scale (N-PASS), a validated tool used to assess pain and sedation in neonates, with scores ranging from − 10 to + 10 [19]. The Turkish version has also been validated [20].

Suction procedures were performed using 6, 8, and 10 French catheters. A centrally maintained and calibrated vacuum aspiration system was used. A 0.9% sodium chloride solution (10 mL ampoule) and 10 mL syringes were utilized. Physiological parameters were monitored via bedside monitors, and suction duration was measured using a stopwatch.

### Outcome measures

Primary and secondary outcomes were predefined in the ClinicalTrials.gov registry (NCT07111611). The primary outcome was the N-PASS pain subscale score measured during suction (range 0–10; higher scores indicate greater pain). The primary outcome assessment was performed within the first minute after initiation of the suction procedure. Secondary outcomes included physiological parameters (SpO_2_, HR, RR) measured during suction and 10 min after suction, and the N-PASS sedation subscale score measured during suction.

### Statistical analysis

Statistical analyses were performed using NCSS 2023 (Number Cruncher Statistical System, Kaysville, Utah, USA). Descriptive statistics were presented as mean ± standard deviation (SD), median (min–max), frequency, and percentage as appropriate. Normality of continuous variables was assessed using the Shapiro–Wilk test and graphical methods. Student’s t-test was used for normally distributed variables, and the Mann–Whitney U test was used for non-normally distributed variables. Categorical variables were compared using the Pearson chi-square test or Fisher–Freeman–Halton exact test when appropriate. Effect sizes for repeated measures were calculated using Kendall’s W, with corresponding 95% confidence intervals. All statistical tests were two-tailed and a *p* value < 0.05 was considered statistically significant.

### Ethical considerations and trial registration

Ethical approval was obtained from the Clinical Research Ethics Committee of Zeynep Kamil Women’s and Children’s Diseases Training and Research Hospital (Approval No: 159). Written informed consent was obtained from the parents of all enrolled infants.

Trial registration: ClinicalTrials.gov (NCT07111611), registered on 08 August 2025 (retrospectively registered).

## Results

**Fig. 1:**
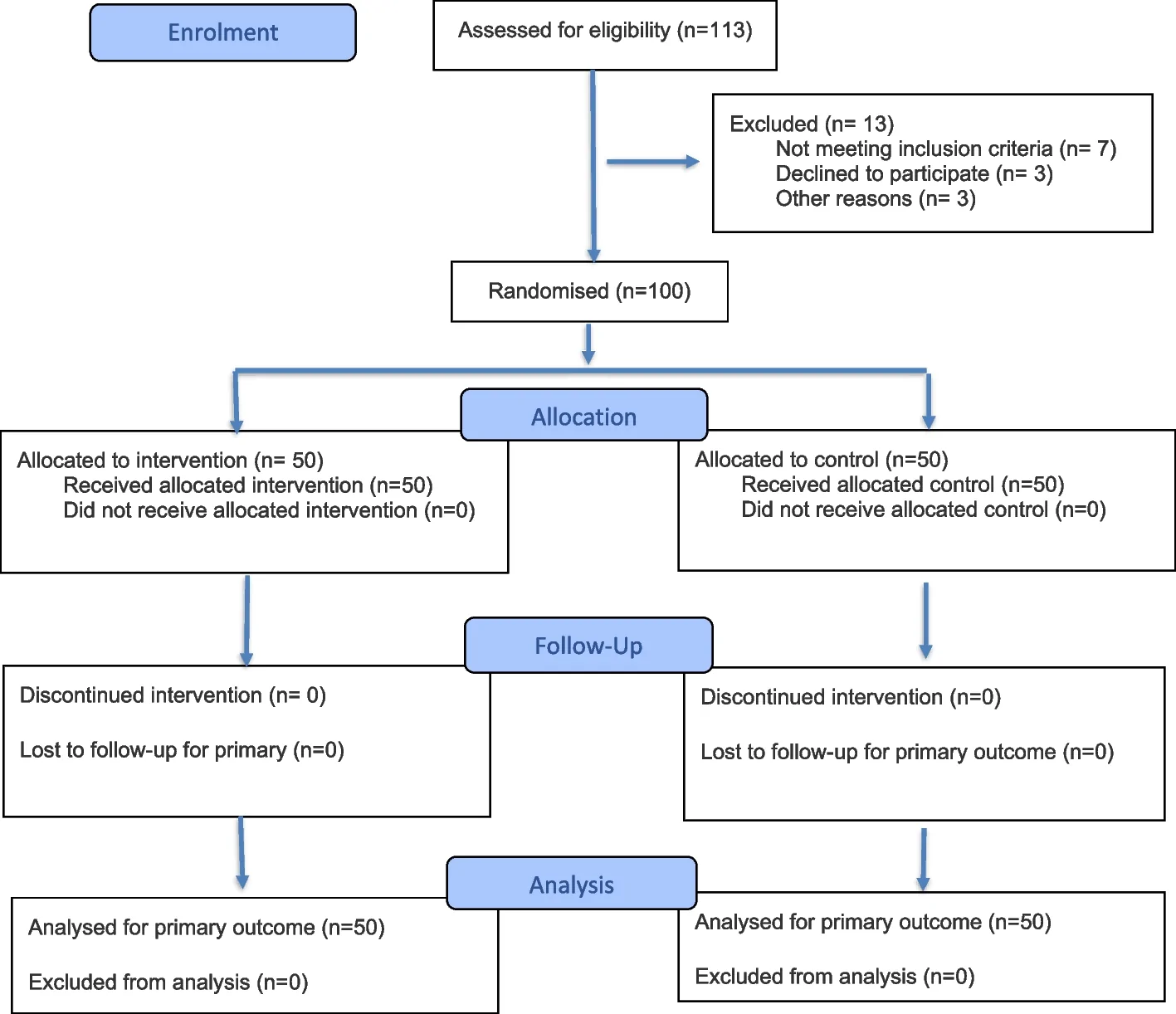
CONSORT flow diagram of participant enrollment, randomization, follow-up, and analysis

A total of 113 preterm infants were assessed for eligibility, of whom 100 were included in the study. Thirteen infants were excluded for various reasons. The CONSORT flow diagram of participant enrollment, randomization, and analysis is presented in Fig. 1.

### Baseline characteristics

A total of 100 preterm infants were included, with 50 in the study group and 50 in the control group. No statistically significant differences were found between the groups in terms of sex, gestational age, birth weight, postnatal age, mechanical ventilation duration, nCPAP initiation day and duration, number of suction days, number of suction procedures, or transition to second-level care (all *p* > 0.05) (Table 1). These findings confirm baseline comparability between groups.

**Table 1:** Baseline demographic and clinical characteristics of the study and control groups

		**Study (*n* = 50)**	**Control (*n* = 50)**	***p-value***
Sex	Female	24 (48%)	25 (50%)	*0.841*
	Male	26 (52%)	25 (50)	
Gestational age (weeks)	*Mean* ± *SD*	32.32 ± 2.70	33.36 ± 2.56	*0.051*
	*Median (Min–Max)*	32 (27–37)	34 (27–37)	
Birth weight (g)	*Mean* ± *SD*	1901.54 ± 588.00	2076.80 ± 678.28	*0.171*
	*Median (Min–Max)*	1865 (867–3000)	2060 (740–3000)	
Postnatal age (days)	*Mean* ± *SD*	3.68 ± 5.48	1.64 ± 2.03	*0.720*
	*Median (Min–Max)*	1 (1–25)	1 (0–11)	
Duration of mechanical ventilation (days)	*Mean* ± *SD*	2.74 ± 6.15	1.54 ± 3.18	*0.146*
	*Median (Min–Max)*	0 (0–33)	0 (0–11)	
Day of nCPAP initiation	*Mean* ± *SD*	3.50 ± 5.87	2.64 ± 3.08	*0.344*
	*Median (Min–Max)*	1 (1–33)	1 (1–12)	
Duration of nCPAP (days)	*Mean* ± *SD*	3.00 ± 1.95	4.12 ± 5.50	*0.771*
	*Median (Min–Max)*	2.5 (1–10)	2 (1–32)	
Number of suction days	*Mean* ± *SD*	3.00 ± 1.70	4.18 ± 5.51	*0.883*
	*Median (Min–Max)*	3 (1–8)	2 (1–32)	
Number of suction procedures	*Mean* ± *SD*	7.72 ± 5.50	12.26 ± 21.68	*0.202*
	*Median (Min–Max)*	6 (1–24)	6 (2–128)	
Day of transition to level II care	*Mean* ± *SD*	6.32 ± 9.50	6.66 ± 7.73	*0.259*
	*Median (Min–Max)*	3 (2–62)	4 (2–43)	

Student’s t-test or Mann–Whitney U test for continuous variables; Pearson chi-square test for categorical variables

^*^p < 0.05, **p < 0.01

### N-PASS pain and sedation scores

Table 2 presents the comparison of N-PASS pain and sedation scores between the study and control groups before suctioning, during the suction procedure, and 10 min after suction.

**Table 2:** Comparison of N-PASS pain and sedation scores before, during, and 10 min after suction

Time	Parameter	Study (*n* = 50)	Control (*n* = 50)	Test statistic/p	Effect size (Kendall’s W)	95% CI
Before suction	*N-PASS Pain*	1.44 ± 1.59	1.82 ± 1.32	*p* = 0.084	0.10	0.01–0.24
	*N-PASS Sedation*	− 1.60 ± 1.51	− 2.12 ± 2.24	Z = 1.96, *p* = 0.050	0.20	0.01–0.39
During suction	*N-PASS Pain*	2.70 ± 2.14	5.28 ± 1.88	**Z = 5.57, *p* < 0.001**	**0.72**	**0.61–0.81**
	*N-PASS Sedation*	− 1.24 ± 1.38	− 1.64 ± 2.16	**Z = 2.23, *p* = 0.026**	**0.28**	**0.16–0.42**
10 min after suction	*N-PASS Pain*	0.46 ± 0.86	1.72 ± 1.34	**Z = 5.26, *p* < 0.001**	**0.72**	**0.61–0.81**
	*N-PASS Sedation*	− 1.24 ± 1.38	− 2.04 ± 1.74	**Z = 3.30, *p* = 0.001**	**0.28**	**0.16–0.42**

^*^Comparisons were performed using the Mann–Whitney U test. Z values, effect sizes (Kendall’s W), and 95% confidence intervals are reported where applicable

^*^p < 0.05, **p < 0.01 Bold values indicate statistically significant results (*p *< 0.05)

Before suctioning, there was no statistically significant difference between the groups in terms of N-PASS pain scores (*p* = 0.084). Sedation scores showed a borderline difference between groups (Z = 1.96, *p* = 0.050).

During suctioning, N-PASS pain scores were significantly higher in the control group than in the study group (Z = 5.57, *p* < 0.001). Ten minutes after suctioning, pain scores remained significantly higher in the control group (Z = 5.26, *p* < 0.001).

The study group demonstrated significantly higher sedation levels both during the suction procedure and 10 min after suctioning compared with the control group (Z = 2.23, *p* = 0.026 and Z = 3.30, *p* = 0.001, respectively).

Changes in N-PASS scores over time demonstrated varying effect sizes. For sedation, small-to-moderate effect sizes were observed (Kendall’s W ranging from 0.16 to 0.28, 95% CI: 0.12–0.42). In contrast, changes in N-PASS pain scores showed large effect sizes in both groups (Kendall’s W ranging from 0.72 to 0.81, 95% CI: 0.61–0.87).

### Physiological parameters

Table 3 presents the comparison of physiological parameters between the study and control groups before suctioning, during the suction procedure, and 10 min after suction.

**Table 3:** Comparison of physiological parameters before, during, and 10 min after suction

Time	Parameter	Study (*n* = 50)	Control (*n* = 50)	Test statistic/p	Effect size (Kendall’s W)	95% CI
Before suction	*HR (Mean* ± *SD)*	138.62 ± 8.64	135.94 ± 10.28	*p* = 0.185	0.08	0.01–0.21
	*RR (Mean* ± *SD)*	40.98 ± 3.24	40.34 ± 3.07	*p* = 0.492	0.04	0.00–0.15
	*SpO₂ (Median [Min–Max])*	95 (92–98)	95 (91–98)	*p* = 0.471	0.05	0.00–0.16
	*Pink (n, %)*	50 (100%)	50 (100%)	–	–	–
During suction	*HR (Mean* ± *SD)*	142.20 ± 12.89	155.18 ± 20.52	**Z = 3.25, *p* = 0.001**	**0.22**	**0.08–0.42**
	*RR (Mean* ± *SD)*	42.50 ± 5.01	42.08 ± 2.68	*p* = 0.421	0.10	0.01–0.32
	*SpO₂ (Median [Min–Max])*	93 (88–98)	89 (82–96)	**Z = 4.75, *p* < 0.001**	**0.54**	**0.34–0.73**
	*Pink (n, %)*	39 (78%)	24 (48%)	**Z = 2.94, *p* = 0.003**	**0.22**	**0.10–0.34**
10 min after suction	*HR (Mean* ± *SD)*	137.12 ± 8.37	141.84 ± 8.92	**Z = 3.08, *p* = 0.002**	**0.22**	**0.08–0.42**
	*RR (Mean* ± *SD)*	40.96 ± 3.49	39.88 ± 2.81	*p* = 0.250	0.10	0.01–0.32
	*SpO₂ (Median [Min–Max])*	96 (93–99)	95 (91–98)	**Z = 3.22, *p* = 0.001**	**0.54**	**0.34–0.73**
	*Pink (n, %)*	50 (100%)	41 (82%)	**Z = 3.13, *p* = 0.002**	**0.22**	**0.10–0.34**

^*^Comparisons were performed using the Mann–Whitney U test for continuous variables and the Fisher–Freeman–Halton test for categorical variables. Z values, effect sizes (Kendall’s W), and 95% confidence intervals are reported where applicable

^*^p < 0.05, **p < 0.01 Bold values indicate statistically significant results (*p *< 0.05)

Before suctioning, there were no statistically significant differences between the groups in heart rate (HR), respiratory rate (RR), or oxygen saturation (SpO_2_) (*p* > 0.05).

During suctioning, HR was significantly higher in the control group compared with the study group (Z = 3.25, *p* = 0.001). This difference remained significant 10 min after suctioning (Z = 3.08, *p* = 0.002). No statistically significant differences were observed between the groups in RR at any measurement time (*p* > 0.05).

In contrast, SpO_2_ values were significantly higher in the study group both during suctioning and 10 min after suction (Z = 4.75, *p* < 0.001 and Z = 3.22, *p* = 0.001, respectively). Similarly, the color parameter showed significant differences between the groups during suctioning and 10 min after suction, with a higher proportion of infants with pink color in the study group (Z = 2.94, *p* = 0.003 and Z = 3.13, *p* = 0.002).

For physiological parameters, heart rate and respiratory rate demonstrated small-to-moderate effect sizes (heart rate: Kendall’s W ranging from 0.22 to 0.47, 95% CI: 0.08–0.71; respiratory rate: W ranging from 0.10 to 0.26, 95% CI: 0.01–0.49). SpO_2_ demonstrated moderate-to-large effect sizes (Kendall’s W ranging from 0.54 to 0.88, 95% CI: 0.34–0.95). Color changes demonstrated small-to-moderate effect sizes (Kendall’s W ranging from 0.22 to 0.39, 95% CI: 0.10–0.52).

### Overall findings

Overall, suctioning was associated with increased pain scores and physiological responses, particularly in the control group. Compared with the control group, infants in the study group had significantly lower N-PASS pain scores and higher sedation scores during suctioning and 10 min after the procedure. In addition, the study group demonstrated more stable physiological parameters, including lower heart rate and higher oxygen saturation during and after suctioning.

## Discussion

In this randomized controlled study, the use of a less invasive suction technique in preterm infants receiving non-invasive ventilation (NIV) was associated with a significant reduction in procedural pain and improved physiological stability. The lower N-PASS pain score observed in the intervention group compared with the control group during suctioning (2 vs. 5) indicates a clinically meaningful reduction in pain.

In addition, infants in the less invasive suction group demonstrated higher sedation scores and more stable cardiorespiratory parameters during the procedure. Effect size analysis showed large effects for pain in both groups, indicating that suctioning induces substantial behavioral responses; however, pain intensity remained lower in the intervention group. Although a universally accepted threshold for clinical significance in N-PASS scores has not been clearly established, the magnitude of the observed difference suggests a clinically meaningful reduction in procedural pain.

In clinical practice, suctioning procedures are largely guided by nursing judgment, including catheter selection, negative pressure levels, and duration of application [3]. The limited availability of evidence-based guidance regarding suction techniques may contribute to variability in clinical practice [3]. From a nursing perspective, suctioning is among the most frequently performed procedures in neonatal intensive care units and is commonly carried out by nurses as part of airway management. In addition to maintaining airway patency, neonatal nurses play an important role in minimizing procedural pain and ensuring comfort in preterm infants. Previous literature indicates that reducing painful stimuli and procedural stress is a key component of developmentally supportive neonatal care [21]. Therefore, the use of less traumatic suction techniques may help reduce procedural discomfort and maintain physiological stability in preterm infants.

Previous randomized studies have mainly focused on adjunctive non-pharmacological interventions, such as swaddling, oropharyngeal colostrum administration, or auricular massage, to reduce suction-related pain [15, 22]. In contrast, oral glucose administration has not consistently demonstrated benefit in this context [23]. These studies primarily evaluated supportive interventions applied during suctioning rather than modifications of the suction technique itself.

Earlier procedural suggestions aimed at reducing mucosal trauma included saline irrigation to decrease catheter use and techniques designed to avoid direct mucosal contact during suctioning [17, 18]. However, these approaches have not been systematically evaluated within randomized controlled study designs. Evidence specifically addressing modifications of suction techniques in NIV-supported preterm infants remains limited. The present study contributes to this area by directly targeting the traumatic component of the suction procedure.

In the present study, saline instillation was performed in small aliquots and not as a single bolus, with simultaneous suction applied to facilitate secretion mobilization. The reported saline volume referred to the cumulative total volume administered throughout the procedure rather than a single instillation. Although the total volume used may appear relatively high, the administration was gradual and guided by the infant’s clinical condition. All infants were continuously monitored during the procedure, and no adverse events such as aspiration or near-drowning effects were observed. This approach is consistent with previously described techniques in the literature [18]. However, given the multi-component nature of the intervention, including differences in catheter size, suction pressure, and saline use, the observed effects cannot be attributed to a single factor. The protocol used in the present study was adapted from the irrigation-assisted, non-contact nasopharyngeal suction technique described by Waisman et al. [18], which recommends a suction pressure of approximately 150 mmHg. Therefore, the term “less invasive” refers primarily to the non-contact nature of the procedure and the avoidance of deep catheter advancement into the airway rather than lower suction pressure alone. Although the intervention utilized a larger catheter diameter and higher suction pressure than the conventional approach, the absence of direct mucosal contact may have reduced mechanical stimulation and mucosal trauma. Nevertheless, the relatively high suction pressure should be interpreted with caution, particularly in preterm infants, and further studies are needed to determine the optimal procedural parameters for this technique.

Consistent with previous reports describing desaturation and increased stress responses during suctioning [1, 7] infants in the conventional suction group in our study exhibited greater physiological fluctuations during the procedure. In contrast, the less invasive suction approach was associated with improved physiological stability. Previous studies have shown that airway suctioning may induce physiological responses in neonates, including changes in heart rate, oxygen saturation, and hemodynamic parameters during the procedure [24, 25]. Similarly, Merter et al. demonstrated that endotracheal suctioning may reduce cerebral oxygenation in preterm infants, highlighting the physiological impact of airway manipulation [26]. These findings support the hypothesis that reducing mechanical stimulation of the airway may contribute to improved physiological stability during suction procedures. Reduced mucosal stimulation during suctioning may limit autonomic activation and thereby decrease cardiorespiratory instability in vulnerable preterm infants. Although no adverse events were observed during the study, the sample size and study design limit definitive conclusions regarding safety.

The use of a relatively higher suction pressure in the intervention group may have contributed to more effective secretion removal and shorter procedure duration. However, suction was applied without direct mucosal contact and for a brief period (5–10 s), which may have minimized potential mechanical trauma. This approach is consistent with previously described techniques in the literature [18] may explain the apparent paradox of higher suction pressure being associated with improved physiological stability.

The effect size analysis suggests that although suctioning induces significant physiological and behavioral (pain-related) responses in both groups, these responses were more pronounced in the control group. In contrast, the less invasive suction protocol was associated with more stable physiological responses and lower pain intensity, indicating a clinically more favorable profile.

Repeated exposure to procedural pain in preterm infants has been associated with adverse neurodevelopmental outcomes [8, 13, 27]. Recent studies also suggest that repeated procedural pain during NICU hospitalization may disrupt brain maturation and growth in preterm infants [28, 29]. Therefore, reducing pain during frequently performed procedures such as suctioning is clinically important and is considered a key component of developmentally supportive neonatal care [30]. The findings of the present study suggest that modifying the suction technique, without eliminating necessary airway clearance, may represent a more stable and developmentally supportive approach in neonatal care. While these findings are promising, they should be interpreted with caution given the study limitations, including the single-center design, lack of blinding, and the multi-component nature of the intervention.

### Limitations

This study has several limitations. It was conducted in a single tertiary care center, which may limit the generalizability of the findings. In addition, extremely preterm infants (< 32 weeks of gestation) were not included, which may limit the generalizability of the findings to this population.

Assessments were performed by three trained research nurses; however, inter-observer reliability was not formally evaluated, which may have influenced measurement consistency. Blinding of care providers and outcome assessors was not feasible due to the nature of the intervention and the clinical setting, which may have introduced potential observer bias. Therefore, the potential influence of bedside nurses on outcome assessment cannot be excluded.

The multi-component nature of the intervention makes it difficult to isolate the individual effects of each component and may have introduced potential confounding. In addition, the lack of adjustment for potential confounders may have influenced the estimation of effect sizes. Although CRIB II scores were available for some participants, complete data were not available for the entire cohort; therefore, these variables were not included in the analyses.

Finally, only short-term physiological and behavioral outcomes were evaluated, and the trial was registered retrospectively, which may limit transparency.

## Conclusion

The less invasive nasopharyngeal suction technique was associated with reduced procedural pain and improved physiological stability in preterm infants receiving non-invasive ventilation. Modifying suction techniques to minimize airway manipulation may contribute to improved physiological stability in routine neonatal care practices. Further multicenter studies with larger samples and long-term follow-up are needed to confirm these findings and evaluate potential neurodevelopmental implications.

## Supplementary Information

Supplementary Material 1.

## Data Availability

The datasets generated and/or analyzed during the current study are available from the corresponding author on reasonable request.

## Abbreviations

NIV: Non-invasive ventilation
NICU: Neonatal intensive care unit
nCPAP: Nasal continuous positive airway pressure
N-PASS: Neonatal Pain, Agitation and Sedation Scale
HR: Heart rate
RR: Respiratory rate
SpO₂: Peripheral oxygen saturation
